# Tighter Constraints of Multi-Qubit Entanglement in Terms of Nonconvex Entanglement Measures LCREN and LCRENoA

**DOI:** 10.3390/e26020127

**Published:** 2024-01-31

**Authors:** Zhongxi Shen, Dongping Xuan, Wen Zhou, Zhixi Wang, Shao-Ming Fei

**Affiliations:** 1 School of Mathematical Sciences, Capital Normal University, Beijing 100048, China; 18738951378@163.com (Z.S.); 2230501014@cnu.edu.cn (D.X.); 2230501027@cnu.edu.cn (W.Z.); wangzhx@cnu.edu.cn (Z.W.); 2 Max-Planck-Institute for Mathematics in the Sciences, 04103 Leipzig, Germany

**Keywords:** monogamy, polygamy, LCREN, LCRENoA

## Abstract

The monogamy property of entanglement is an intriguing feature of multipartite quantum entanglement. Most entanglement measures satisfying the monogamy inequality have turned out to be convex. Whether nonconvex entanglement measures obey the monogamy inequalities remains less known at present. As a well-known measure of entanglement, the logarithmic negativity is not convex. We elucidate the constraints of multi-qubit entanglement based on the logarithmic convex-roof extended negativity (LCREN) and the logarithmic convex-roof extended negativity of assistance (LCRENoA). Using the Hamming weight derived from the binary vector associated with the distribution of subsystems, we establish monogamy inequalities for multi-qubit entanglement in terms of the αth-power (α≥4ln2) of LCREN, and polygamy inequalities utilizing the αth-power (0≤α≤2) of LCRENoA. We demonstrate that these inequalities give rise to tighter constraints than the existing ones. Furthermore, our monogamy inequalities are shown to remain valid for the high-dimensional states that violate the CKW monogamy inequality. Detailed examples are presented to illustrate the effectiveness of our results in characterizing the multipartite entanglement distributions.

## 1. Introduction

Quantum entanglement is vital in quantum mechanics, offering profound insights into the essence of quantum correlations and serving pivotal functions in quantum information processing. As the key resource in quantum tasks, quantum entanglement has been used in many quantum communication protocols such as superdense coding [1], quantum cryptography [2], quantum teleportation [3] and remote-state preparation [4]. One remarkable feature of quantum entanglement, setting it apart from classical correlations, is its inherent limitation in being shared among multipartite quantum systems, referred to as the monogamy of entanglement (MoE) [5,6]. MoE’s restrictions on the information accessible to potential eavesdroppers regarding secret key extraction play a pivotal role in the security of many information-theoretic protocols such as quantum key distribution [7,8,9]. MoE has been widely used in many areas of physics such as condensed-matter physics [10] and even black-hole physics [11]. MoE may also help to investigate the efficiency of entanglement used in quantum cryptography and in characterizations of the entanglement distributions.

Coffman, Kundu and Wootters (CKW) firstly characterized the monogamy of entanglement for three-qubit states $\rho_{ABC}$,

(1) $$\tau \left(\rho_{A|BC}\right)\geq \tau \left(\rho_{A|B}\right)+\tau \left(\rho_{A|C}\right),$$

where $\rho_{AB}={\mathrm{tr}}_{C}\left(\rho_{ABC}\right)$ and $\rho_{AC}={\mathrm{tr}}_{B}\left(\rho_{ABC}\right)$ are the reduced density matrices by tracing over the subsystem *C* and *B*, respectively, with ${\mathrm{tr}}_{B}$ (${\mathrm{tr}}_{C}$) denoting the partial trace with respect to the subsystem *B* (*C*). $\tau \left(\rho_{A|BC}\right)$ is the tangle of $\rho_{ABC}$ between subsystems *A* and BC, and $\tau \left(\rho_{A|B}\right)$ ($\tau \left(\rho_{A|C}\right)$) is the tangle between *A* and *B* (*A* and *C*) [12]. The CKW inequality illustrates the incompatibility of the two-qubit entanglement between $\tau \left(\rho_{A|B}\right)$ and $\tau \left(\rho_{A|C}\right)$. Thus, the sum of the entanglement of the two-qubit systems cannot surpass the collective entanglement between *A* and BC. The CKW inequality was expanded to encompass different measures of bipartite entanglement, enabling its extension to multi-qubit systems [13] and higher-dimensional quantum systems [14,15,16,17].

The entanglement of assistance, as a complementary quantity to bipartite entanglement, holds immense significance too. It notably displays a dualistic monogamous nature within multi-qubit quantum systems and gives rise to polygamous relationships. Whereas MoE inequalities provide upper bounds on the shareability of entanglement among quantum subsystems, the authors in Ref. [18] prove that this bound also acts as a lower bound (conjectured in Ref. [19]) for the distribution of entanglement, the entanglement of assistance, to a target pair of *A* and *B* [20,21,22]. This distribution of entanglement is established by performing collective operations on the rest of the subsystems so as to maximize the shared entanglement between *A* and *B*. The duality of entanglement shareability versus entanglement of assistance is evident in that the upper bound for the former is the lower bound for the latter. For a three-qubit state $\rho_{ABC}$, the polygamy inequality is defined by

$$\tau^{a}\left(\rho_{A|BC}\right)\leq \tau^{a}\left(\rho_{A|B}\right)+\tau^{a}\left(\rho_{A|C}\right),$$

where $\tau^{a}\left(\rho_{A|BC}\right)$ is the tangle of assistance [18,19]. The polygamy inequality was extended to encompass multi-qubit systems and certain classes of higher-dimensional quantum systems by using diverse entropic entanglement measures [16,23,24,25,26].

Lately, there have been noteworthy discussions on monogamy and polygamy inequalities based on the αth-power of entanglement measures [27,28,29]. In Ref. [30], Kim derived strict monogamy inequalities by using the Hamming weight. This approach effectively captures the entangled nature of quantum states and offers a novel study of monogamy. Subsequently, comprehensive sets of inequalities associated with the Hamming weight of entanglement measures have been introduced [31,32].

It is generally acknowledged that monogamy inequalities are consistently fulfilled by entanglement measures exhibiting convexity. The question of whether a nonconvex measure of entanglement abides by the monogamy inequality remains less known presently. The nonconvex nature of the logarithmic negativity is surprising, as it is generally considered that convexity describes the local physical process of losing information [33,34]. However, it should be noted that the convexity is primarily a mathematical requirement for entanglement monotones and does not necessarily describe a physical process that involves the loss of information about a quantum system [34]. Indeed, it is the combination of concavity and the monotonicity of the logarithm that allows for the proof of the nonincreasing of the logarithmic negativity under positive partial transpose (PPT)-preserving operations [34]. In addition, the logarithmic negativity, which possesses an operational interpretation, is an entanglement monotone under both general local operations and classical communication (LOCC) [35]. The measure is the upper bound on distillable entanglement [34,35] and is related to the entanglement cost under PPT-preserving operations [35]. Therefore, complementary to those of the convex measures of entanglement, the monogamy of logarithmic negativity is a key issue in the theory of quantum entanglement.

In this paper, we offer a more detailed characterization of multi-qubit entanglement by using these nonconvex entanglement measures. Our research reveals that the monogamy and polygamy inequalities we present are upheld in a tighter way compared to those elucidated in Ref. [36]. Additionally, the newly proposed monogamy inequalities are shown to be more effective in addressing counterexamples raised by the CKW monogamy inequality in higher-dimensional systems.

## 2. Preliminaries

Let us first review the conceptions of LCREN and LCRENoA, as well as the monogamy and polygamy inequalities associated with them in multi-qubit systems. For a quantum state $\rho_{AB}$ on Hilbert space $H_{A}⊗H_{B}$, the negativity $N(\rho_{AB})$ is defined as [33,34,37]

$$N\left(\rho_{AB}\right)={∥\rho_{AB}^{T_{A}}∥}_{1}-1,$$

where $\rho_{AB}^{T_{A}}$ denotes the partial transpose of $\rho_{AB}$ with respect to the subsystem *A*, and the trace norm ${∥X∥}_{1}$ of any *X* is defined by ${∥X∥}_{1}=\mathrm{tr}\sqrt{XX^{†}}$. The logarithmic negativity is defined as [33,34],

$$E_{N}\left(\rho_{AB}\right)=\log_{2}{∥\rho_{AB}^{T_{A}}∥}_{1}=\log_{2}\left[N\left(\rho_{AB}\right)+1\right].$$

This measure serves as an entanglement monotone under both general local operation and classical communication (LOCC), as well as positive partial transpose (PPT)-preserving operations. It is also additive in nature but lacks convexity [34].

The convex-roof extended negativity (CREN) of a bipartite state $\rho_{AB}$ is defined by [38],

$$\tilde{N}\left(\rho_{AB}\right)=\min_{{\{p_{k},|\varphi_{k}\rangle }_{AB}\}}\sum_{k}p_{k}N{(|\varphi_{k}\rangle }_{AB}),$$

and the CREN of assistance (CRENoA) is defined by [14],

$${\tilde{N}}_{a}\left(\rho_{AB}\right)=\max_{{\{p_{k},|\varphi_{k}\rangle }_{AB}\}}\sum_{k}p_{k}N{(|\varphi_{k}\rangle }_{AB}),$$

where the minimum and maximum are taken over all possible pure-state decompositions of $\rho_{AB}=\sum_{k}p_{k}|\varphi_{k}\rangle_{AB}\langle \varphi_{k}|$ with $p_{k}\geq 0$, and $\sum_{k}p_{k}=1$. By definition, both CREN and CRENoA of a pure state are equal to the negativity.

For any bipartite state $\rho_{AB}$, the LCREN is defined by

(2) $$E_{\tilde{N}}\left(\rho_{AB}\right)=\log_{2}\left[\tilde{N}\left(\rho_{AB}\right)+1\right].$$

LCREN is a bona fide measure of entanglement. It exhibits both a nonincrease under LOCC and an average nonincrease under LOCC, which can be attributed to CREN’s entanglement monotonicity, logarithmic monotonicity and logarithm concavity. Nonetheless, similar to the logarithmic negativity, LCREN lacks convexity.

For any (N+1)-qubit state $\rho_{AB_{0}\cdots B_{N-1}}$, a monogamy inequality has been presented in Ref. [36] for α≥4ln2,

(3) $$E_{\tilde{N}}^{\alpha }\left(\rho_{A|B_{0}\cdots B_{N-1}}\right)\geq \sum_{i=0}^{N-1}E_{\tilde{N}}^{\alpha }\left(\rho_{A|B_{i}}\right),$$

where $E_{\tilde{N}}\left(\rho_{A|B_{0}\cdots B_{N-1}}\right)$ is the LCREN of $\rho_{AB_{0}\cdots B_{N}-1}$ with respect to the bipartition *A* and $B_{0}\cdots B_{N-1}$, and $E_{\tilde{N}}\left(\rho_{A|B_{i}}\right)$ is the LCREN of the reduced density matrix $\rho_{AB_{i}}$, i=0,⋯,N−1.

Similar to LCREN, the LCRENoA is defined by

(4) $$E_{{\tilde{N}}_{a}}\left(\rho_{AB}\right)=\log_{2}\left[{\tilde{N}}_{a}\left(\rho_{AB}\right)+1\right].$$

For 0≤α≤2, we obtain the polygamy inequality [36],

(5) $$E_{{\tilde{N}}_{a}}^{\alpha }\left(\rho_{A|B_{0}\cdots B_{N-1}}\right)\leq \sum_{i=0}^{N-1}E_{{\tilde{N}}_{a}}^{\alpha }\left(\rho_{A|B_{i}}\right),$$

where $E_{{\tilde{N}}_{a}}\left(\rho_{A|B_{0}\cdots B_{N-1}}\right)$ is the LCRENoA of $\rho_{AB_{0}\cdots B_{N}-1}$ with respect to the bipartition *A* and $B_{0}\cdots B_{N-1}$, and $E_{{\tilde{N}}_{a}}\left(\rho_{A|B_{i}}\right)$ is the LCRENoA of the reduced density matrices $\rho_{AB_{i}}$, i=0,⋯,N−1.

The tighter monogamy relations give rise to a more refined characterization of the entanglement distributions among the quantum systems, and significant applications such as in the security of quantum cryptographic protocols based on entanglement by limiting eavesdropper correlations with the honest parties. Therefore, new tighter monogamy relations of entanglement provide better understanding and applications of quantum entanglement. In the forthcoming sections, we present improvements to the above inequalities, achieving significantly tighter constraints on multi-qubit entanglement distribution under specific conditions.

## 3. Tighter Monogamy Inequalities of Multi-Qubit LCREN

We first present a series of notations and definitions to assist in understanding the subsequent discussion. For any nonnegative integer *j* with binary expansion $j=\sum_{i=0}^{N-1}j_{i}2^{i}$, where $\log_{2}j\leq N$ and $j_{i}\in \left\{0,1\right\}$ for i=0,⋯,N−1, one defines a unique binary vector associated with *j*, $\vec{j}=\left(j_{0},\;j_{1},\;\cdots ,j_{N-1}\right)$. The Hamming weight $\omega_{H}\left(\vec{j}\right)$ of the binary vector $\vec{j}$ is defined as the number of 1s in its coordinates [39]. Moreover, the Hamming weight $\omega_{H}\left(\vec{j}\right)$ is bounded above by $\log_{2}j$,

(6) $$\omega_{H}\left(\vec{j}\right)\leq \log_{2}j\leq j.$$

We also require the subsequent lemma, which can be easily verified.

**Lemma 1.**  *For 0≤x≤1 and nonnegative real number α, we have*

(7) $$\begin{array}{c} {\left(1+x\right)}^{\alpha }\geq 1+\alpha x^{\alpha } \end{array}$$

*for α≥1, and*

(8) $$\begin{array}{c} {\left(1+x\right)}^{\alpha }\leq 1+\alpha x^{\alpha } \end{array}$$

*for 0≤α≤1.*

In the subsequent discussion, we present a new class of monogamy relations in terms of the αth-power of LCREN by incorporating the concept of Hamming weight.

**Theorem 1.**  *For any (N+1)-qubit state $\rho_{AB_{0}\ldots B_{N-1}}$, we have*

(9) $${\left[E_{\tilde{N}}\left(\rho_{A|B_{0}B_{1}\ldots B_{N-1}}\right)\right]}^{\alpha }\geq \sum_{j=0}^{N-1}{\left(\frac{\alpha }{4\ln2}\right)}^{\omega_{H}\left(\vec{j}\right)}{\left[E_{\tilde{N}}\left(\rho_{A|B_{j}}\right)\right]}^{\alpha },$$

*where α≥4ln2, $\vec{j}=\left(j_{0},\cdots ,j_{N-1}\right)$ is the vector from the binary representation of j, and $\omega_{H}\left(\vec{j}\right)$ is the Hamming weight of $\vec{j}$.*

**Proof.**  From inequality (3), one has $E_{\tilde{N}}^{4\ln2}\left(\rho_{A|B_{0}\cdots B_{N-1}}\right)\geq \sum_{i=0}^{N-1}E_{\tilde{N}}^{4\ln2}\left(\rho_{A|B_{i}}\right)$. Thus, it is sufficient to show that

(10) $${\left[\sum_{j=0}^{N-1}E_{\tilde{N}}^{4\ln2}\left(\rho_{A|B_{j}}\right)\right]}^{\frac{\alpha }{4\ln2}}\geq \sum_{j=0}^{N-1}{\left(\frac{\alpha }{4\ln2}\right)}^{\omega_{H}\left(\vec{j}\right)}{\left[E_{\tilde{N}}\left(\rho_{A|B_{j}}\right)\right]}^{\alpha }.$$

We can assume, without loss of generality, that the qubit subsystems $B_{0},\ldots ,B_{N-1}$ are appropriately labeled such that

(11) $$E_{\tilde{N}}^{4\ln2}\left(\rho_{A|B_{j}}\right)\geq E_{\tilde{N}}^{4\ln2}\left(\rho_{A|B_{j+1}}\right)\geq 0$$

for j=0,1,…,N−2.

Initially, we demonstrate the validity of inequality (10) for the case of $N=2^{n}$. For n=1, we obtain

(12) $${\left[E_{\tilde{N}}^{4\ln2}\left(\rho_{A|B_{0}}\right)+E_{\tilde{N}}^{4\ln2}\left(\rho_{A|B_{1}}\right)\right]}^{\frac{\alpha }{4\ln2}}={\left[E_{\tilde{N}}\left(\rho_{A|B_{0}}\right)\right]}^{\alpha }{\left(1+\frac{E_{\tilde{N}}^{4\ln2}\left(\rho_{A|B_{1}}\right)}{E_{\tilde{N}}^{4\ln2}\left(\rho_{A|B_{0}}\right)}\right)}^{\frac{\alpha }{4\ln2}}.$$

Combining (7) and (11), we have

(13) $${\left(1+\frac{E_{\tilde{N}}^{4\ln2}\left(\rho_{A|B_{1}}\right)}{E_{\tilde{N}}^{4\ln2}\left(\rho_{A|B_{0}}\right)}\right)}^{\frac{\alpha }{4\ln2}}\geq 1+\frac{\alpha }{4\ln2}{\left(\frac{E_{\tilde{N}}\left(\rho_{A|B_{1}}\right)}{E_{\tilde{N}}\left(\rho_{A|B_{0}}\right)}\right)}^{\alpha }.$$

From (12) and (13), we obtain

$${\left[E_{\tilde{N}}^{4\ln2}\left(\rho_{A|B_{0}}\right)+E_{\tilde{N}}^{4\ln2}\left(\rho_{A|B_{1}}\right)\right]}^{\frac{\alpha }{4\ln2}}\geq {\left[E_{\tilde{N}}\left(\rho_{A|B_{0}}\right)\right]}^{\alpha }+\frac{\alpha }{4\ln2}{\left[E_{\tilde{N}}\left(\rho_{A|B_{1}}\right)\right]}^{\alpha }.$$

Therefore, inequality (10) holds for n=1.

Assuming the validity of inequality (10) for $N=2^{n-1}$ (where n≥2), we now proceed to prove its applicability to the case of $N=2^{n}$. For an (N+1)-qubit state $\rho_{AB_{0}B_{1}\cdots B_{N-1}}$ with its two-qubit reduced density matrices $\rho_{AB_{j}}$ with j=0,⋯,N−1, we have

(14) $$\begin{array}{c} {\left(\sum_{j=0}^{N-1}E_{\tilde{N}}^{4\ln2}\left(\rho_{A|B_{j}}\right)\right)}^{\frac{\alpha }{4\ln2}}={\left(\sum_{j=0}^{2^{n-1}-1}E_{\tilde{N}}^{4\ln2}\left(\rho_{A|B_{j}}\right)\right)}^{\frac{\alpha }{4\ln2}}{\left(1+\frac{\sum_{j=2^{n-1}}^{2^{n}-1}E_{\tilde{N}}^{4\ln2}\left(\rho_{A|B_{j}}\right)}{\sum_{j=0}^{2^{n-1}-1}E_{\tilde{N}}^{4\ln2}\left(\rho_{A|B_{j}}\right)}\right)}^{\frac{\alpha }{4ln2}}. \end{array}$$

Inequality (11) implies that

$$0\leq \frac{\sum_{j=2^{n-1}}^{2^{n}-1}E_{\tilde{N}}^{4\ln2}\left(\rho_{A|B_{j}}\right)}{\sum_{j=0}^{2^{n-1}-1}E_{\tilde{N}}^{4\ln2}\left(\rho_{A|B_{j}}\right)}\leq 1.$$

Thus, Equation (14) and inequality (7) lead to

$${\left(\sum_{j=0}^{N-1}E_{\tilde{N}}^{4\ln2}\left(\rho_{A|B_{j}}\right)\right)}^{\frac{\alpha }{4\ln2}}\geq {\left(\sum_{j=0}^{2^{n-1}-1}E_{\tilde{N}}^{4\ln2}\left(\rho_{A|B_{j}}\right)\right)}^{\frac{\alpha }{4\ln2}}+\frac{\alpha }{4\ln2}{\left(\sum_{j=2^{n-1}}^{2^{n}-1}E_{\tilde{N}}^{4\ln2}\left(\rho_{A|B_{j}}\right)\right)}^{\frac{\alpha }{4\ln2}}.$$

According to the induction hypothesis, we obtain

$${\left(\sum_{j=0}^{2^{n-1}-1}E_{\tilde{N}}^{4\ln2}\left(\rho_{A|B_{j}}\right)\right)}^{\frac{\alpha }{4\ln2}}\geq \sum_{j=0}^{2^{n-1}-1}{\left(\frac{\alpha }{4\ln2}\right)}^{\omega_{H}\left(\vec{j}\right)}{\left[E_{\tilde{N}}\left(\rho_{A|B_{j}}\right)\right]}^{\alpha }.$$

By relabeling the subsystems, the induction hypothesis leads to

$${\left(\sum_{j=2^{n-1}}^{2^{n}-1}E_{\tilde{N}}^{4\ln2}\left(\rho_{A|B_{j}}\right)\right)}^{\frac{\alpha }{4\ln2}}\geq \sum_{j=2^{n-1}}^{2^{n}-1}{\left(\frac{\alpha }{4\ln2}\right)}^{\omega_{H}\left(\vec{j}\right)-1}{\left[E_{\tilde{N}}\left(\rho_{A|B_{j}}\right)\right]}^{\alpha }.$$

Therefore, we have

$${\left(\sum_{j=0}^{2^{n}-1}E_{\tilde{N}}^{4\ln2}\left(\rho_{A|B_{j}}\right)\right)}^{\frac{\alpha }{4\ln2}}\geq \sum_{j=0}^{2^{n}-1}{\left(\frac{\alpha }{4\ln2}\right)}^{\omega_{H}\left(\vec{j}\right)}{\left[E_{\tilde{N}}\left(\rho_{A|B_{j}}\right)\right]}^{\alpha }.$$

Considering the existence of a positive integer *N* satisfying the condition $0\leq N\leq 2^{n}$. Let us now contemplate a pure state consisting of $\left(2^{n}+1\right)$ qubits, $\Gamma_{AB_{0}B_{1}\ldots B_{2^{n}-1}}=\rho_{AB_{0}B_{1}\ldots B_{N-1}}⊗\sigma_{B_{N}\ldots B_{2^{n}-1}}$; the state can be precisely expressed as the tensor product of $\rho_{AB_{0}B_{1}\ldots B_{N-1}}$ and $\sigma_{B_{N}B_{N+1}\ldots B_{2^{n}-1}}$, where $\sigma_{B_{N}B_{N+1}\ldots B_{2^{n}-1}}$ represents an arbitrary $\left(2^{n}-N\right)$-qubit state. We have

$${\left[E_{\tilde{N}}^{4\ln2}\left(\Gamma_{A|B_{0}B_{1}\ldots B_{2^{n}-1}}\right)\right]}^{\frac{\alpha }{4\ln2}}\geq \sum_{j=0}^{2^{n}-1}{\left(\frac{\alpha }{4\ln2}\right)}^{\omega_{H}\left(\vec{j}\right)}{\left[E_{\tilde{N}}\left(\Gamma_{A|B_{j}}\right)\right]}^{\alpha },$$

where $\Gamma_{A|B_{j}}$ denotes the two-qubit reduced density matrix derived from $\Gamma_{AB_{0}B_{1}\ldots B_{2^{n}-1}}$, $j=0,1,\ldots ,2^{n}-1$. Therefore,

$$\begin{array}{cc} {\left[E_{\tilde{N}}^{4\ln2}\left(\rho_{A|B_{0}B_{1}\ldots B_{N-1}}\right)\right]}^{\frac{\alpha }{4\ln2}}= & {\left[E_{\tilde{N}}^{4\ln2}\left(\Gamma_{A|B_{0}B_{1}\ldots B_{2^{n}-1}}\right)\right]}^{\frac{\alpha }{4\ln2}}\\ \geq & \sum_{j=0}^{2^{n}-1}{\left(\frac{\alpha }{4\ln2}\right)}^{\omega_{H}\left(\vec{j}\right)}{\left[E_{\tilde{N}}\left(\Gamma_{A|B_{j}}\right)\right]}^{\alpha }\\ = & \sum_{j=0}^{N-1}{\left(\frac{\alpha }{4\ln2}\right)}^{\omega_{H}\left(\vec{j}\right)}{\left[E_{\tilde{N}}\left(\rho_{A|B_{j}}\right)\right]}^{\alpha }, \end{array}$$

where $\Gamma_{A|B_{0}B_{1}\ldots B_{2^{n}-1}}$ denotes the state under bipartition $AB_{0}\ldots B_{N-1}$ and $B_{N}\ldots B_{2^{n}-1}$, $E_{\tilde{N}}\left(\Gamma_{A|B_{0}B_{1}\cdots B_{2^{n}-1}}\right)=E_{\tilde{N}}\left(\rho_{A|B_{0}B_{1}\cdots B_{N-1}}\right)$, $E_{\tilde{N}}\left(\Gamma_{A|B_{j}}\right)=0$ for $j=N,\cdots ,2^{n}-1$, and $\Gamma_{A|B_{j}}=\rho_{A|B_{j}}$ for each j=0,⋯,N−1, which completes the proof. □

**Remark 1.**  *Since ${\left(\frac{\alpha }{4\ln2}\right)}^{\omega_{H}\left(\vec{j}\right)}⩾1$ for any α≥4ln2, for any (N+1)-qubit state $\rho_{AB_{0}B_{1}\cdots B_{N-1}}$, we can express it using the following relation,*

$${\left[E_{\tilde{N}}\left(\rho_{A|B_{0}B_{1}\ldots B_{N-1}}\right)\right]}^{\alpha }\geq \sum_{j=0}^{N-1}{\left(\frac{\alpha }{4\ln2}\right)}^{\omega_{H}\left(\vec{j}\right)}{\left[E_{\tilde{N}}\left(\rho_{A|B_{j}}\right)\right]}^{\alpha }\geq \sum_{j=0}^{N-1}{\left[E_{\tilde{N}}\left(\rho_{A|B_{j}}\right)\right]}^{\alpha }.$$

*Therefore, our inequality (9) in Theorem 1 is always tighter than the inequality (3) in Ref. [36].*

**Example 1.**  *In the generalized Schmidt decomposition, the three-qubit state ${|\phi \rangle }_{ABC}$ can be expressed as [38,40],*

(15) $${|\phi \rangle }_{ABC}=\lambda_{0}|000\rangle +\lambda_{1}e^{i\varphi }|100\rangle +\lambda_{2}|101\rangle +\lambda_{3}|110\rangle +\lambda_{4}|111\rangle ,$$

**Figure 1:**
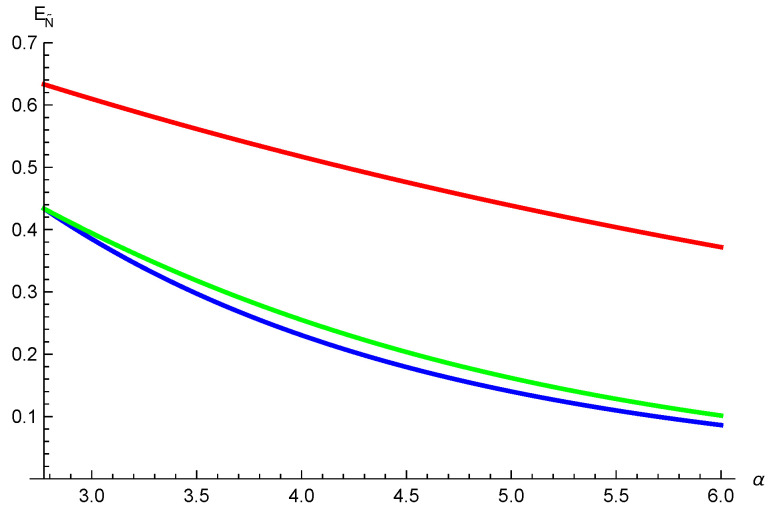
The red line is the exact values of $E_{\tilde{N}}\left(\rho_{A|BC}\right)$. The green and blue lines represent the lower bounds from our result (9) and the result (3) in [36], respectively.

*where $\lambda_{i}\geq 0$, i=0,1,⋯,4, and $\sum_{i=0}^{4}\lambda_{i}^{2}=1$. One obtains $\tilde{N}\left(\rho_{A|BC}\right)=2\lambda_{0}\sqrt{\lambda_{2}^{2}+\lambda_{3}^{2}+\lambda_{4}^{2}}$, $\tilde{N}\left(\rho_{A|B}\right)=2\lambda_{0}\lambda_{2}$ and $\tilde{N}\left(\rho_{A|C}\right)=2\lambda_{0}\lambda_{3}$. Setting $\lambda_{0}=\lambda_{3}=\lambda_{4}=1/\sqrt{5}$, $\lambda_{2}=\sqrt{2/5}$ and $\lambda_{1}=0$, we have $\tilde{N}\left(\rho_{A|BC}\right)=4/5$, $\tilde{N}\left(\rho_{A|B}\right)=2\sqrt{2}/5$ and $\tilde{N}\left(\rho_{A|C}\right)=2/5$. Using relation (2) we have $E_{\tilde{N}}\left(\rho_{A|BC}\right)=\log_{2}\frac{9}{5}$, $E_{\tilde{N}}\left(\rho_{A|B}\right)=\log_{2}\left(2\sqrt{2}/5+1\right)$ and $E_{\tilde{N}}\left(\rho_{A|C}\right)=\log_{2}\frac{7}{5}$. Thus, ${\left[E_{\tilde{N}}\left(\rho_{A|BC}\right)\right]}^{\alpha }\geq {\left(\log_{2}\left(2\sqrt{2}/5+1\right)\right)}^{\alpha }+\frac{\alpha }{4\ln2}{\left(\log_{2}\frac{7}{5}\right)}^{\alpha }$ from our result (9), and ${\left[E_{\tilde{N}}\left(\rho_{A|BC}\right)\right]}^{\alpha }\geq {\left(\log_{2}\left(2\sqrt{2}/5+1\right)\right)}^{\alpha }+{\left(\log_{2}\frac{7}{5}\right)}^{\alpha }$ from the result (3) given in Ref. [36]. One can see our inequality (9) is tighter than the result (3) in Ref. [36] for α≥4ln2, see Figure 1.*

Inequality (9) can be further enhanced under specific circumstances, resulting in a significantly tightened form.

**Theorem 2.**  *For α≥4ln2, any (N+1)-qubit state $\rho_{AB_{0}\ldots B_{N-1}}$ satisfies*

(16) $${\left[E_{\tilde{N}}\left(\rho_{A|B_{0}B_{1}\ldots B_{N-1}}\right)\right]}^{\alpha }\geq \sum_{j=0}^{N-1}{\left(\frac{\alpha }{4\ln2}\right)}^{j}{\left[E_{\tilde{N}}\left(\rho_{A|B_{j}}\right)\right]}^{\alpha },$$

*if*

$$E_{\tilde{N}}^{4\ln2}\left(\rho_{A|B_{i}}\right)\geq \sum_{j=i+1}^{N-1}E_{\tilde{N}}^{4\ln2}\left(\rho_{A|B_{j}}\right)$$

*for i=0,1,…,N−2.*

**Proof.**  From inequality (3), we only need to prove

(17) $${\left[\sum_{j=0}^{N-1}E_{\tilde{N}}^{4\ln2}\left(\rho_{A|B_{j}}\right)\right]}^{\frac{\alpha }{4\ln2}}\geq \sum_{j=0}^{N-1}{\left(\frac{\alpha }{4\ln2}\right)}^{j}{\left[E_{\tilde{N}}\left(\rho_{A|B_{j}}\right)\right]}^{\alpha }.$$

Here, we employ mathematical induction on *N*. It becomes evident that inequality (17) is valid for N=2, as derived from (9), assuming its validity for all positive integers smaller than *N*. Since $\frac{\sum_{j=i+1}^{N-1}E_{\tilde{N}}^{4\ln2}\left(\rho_{A|B_{j}}\right)}{E_{\tilde{N}}^{4\ln2}\left(\rho_{A|B_{i}}\right)}⩽1$, we have

$$\begin{array}{ccc} {\left[\sum_{j=0}^{N-1}E_{\tilde{N}}^{4\ln2}\left(\rho_{A|B_{j}}\right)\right]}^{\frac{\alpha }{4\ln2}} & = & E_{\tilde{N}}^{\alpha }\left(\rho_{A|B_{0}}\right){\left(1+\frac{\sum_{j=1}^{N-1}E_{\tilde{N}}^{4\ln2}\left(\rho_{A|B_{j}}\right)}{E_{\tilde{N}}^{4\ln2}\left(\rho_{A|B_{0}}\right)}\right)}^{\frac{\alpha }{4\ln2}}\\ & ⩾ & E_{\tilde{N}}^{\alpha }\left(\rho_{A|B_{0}}\right)\left[1+\frac{\alpha }{4\ln2}{\left(\frac{\sum_{j=1}^{N-1}E_{\tilde{N}}^{4\ln2}\left(\rho_{A|B_{j}}\right)}{E_{\tilde{N}}^{4\ln2}\left(\rho_{A|B_{0}}\right)}\right)}^{\frac{\alpha }{4\ln2}}\right]\\ & = & E_{\tilde{N}}^{\alpha }\left(\rho_{A|B_{0}}\right)+\frac{\alpha }{4\ln2}{\left(\sum_{j=1}^{N-1}E_{\tilde{N}}^{4\ln2}\left(\rho_{A|B_{j}}\right)\right)}^{\frac{\alpha }{4\ln2}}\\ & ⩾ & E_{\tilde{N}}^{\alpha }\left(\rho_{A|B_{0}}\right)+\frac{\alpha }{4\ln2}\sum_{j=1}^{N-1}{\left(\frac{\alpha }{4\ln2}\right)}^{j-1}E_{\tilde{N}}^{\alpha }\left(\rho_{A|B_{j}}\right)\\ & = & \sum_{j=0}^{N-1}{\left(\frac{\alpha }{4\ln2}\right)}^{j}{\left[E_{\tilde{N}}\left(\rho_{A|B_{j}}\right)\right]}^{\alpha }, \end{array}$$

where the first inequality stems from Lemma 1, and the subsequent inequality relies on the induction hypothesis. □

**Remark 2.**  *According to (6), for any α≥4ln2 one has*

$$\begin{array}{cc} {\left[E_{\tilde{N}}\left(\rho_{A|B_{0}\ldots B_{N-1}}\right)\right]}^{\alpha }\geq & \sum_{j=0}^{N-1}{\left(\frac{\alpha }{4\ln2}\right)}^{j}{\left(E_{\tilde{N}}\left(\rho_{A|B_{j}}\right)\right)}^{\alpha }\\ \geq & \sum_{j=0}^{N-1}{\left(\frac{\alpha }{4\ln2}\right)}^{\omega_{H}\left(\vec{j}\right)}{\left[E_{\tilde{N}}\left(\rho_{A|B_{j}}\right)\right]}^{\alpha }. \end{array}$$

*Hence, within specific conditions, the inequality (16) derived from Theorem 2 exhibits as being tighter compared to the inequality (9) established in Theorem 1.*

**Example 2.**  *Let us consider the four-qubit entangled decoherence-free state [41], $\left|\Phi \rangle =a\right|\Psi_{0}\rangle +b|\Psi_{1}\rangle$, where $|\Psi_{i}\rangle$ are logic basis states given by*

(18) $$\begin{array}{ccc} |\Psi_{0}\rangle_{ABCD} & = & \frac{1}{2}(|01\rangle -{\left|10\rangle \right)}_{AB}(|01\rangle -{\left|10\rangle \right)}_{CD},\\ |\Psi_{1}\rangle_{ABCD} & = & \frac{1}{2\sqrt{3}}(2|1100\rangle +2|0011\rangle -|1010\rangle -|1001\rangle \\ & & -|0101\rangle -{\left|0110\rangle \right)}_{ABCD}. \end{array}$$

**Figure 2:**
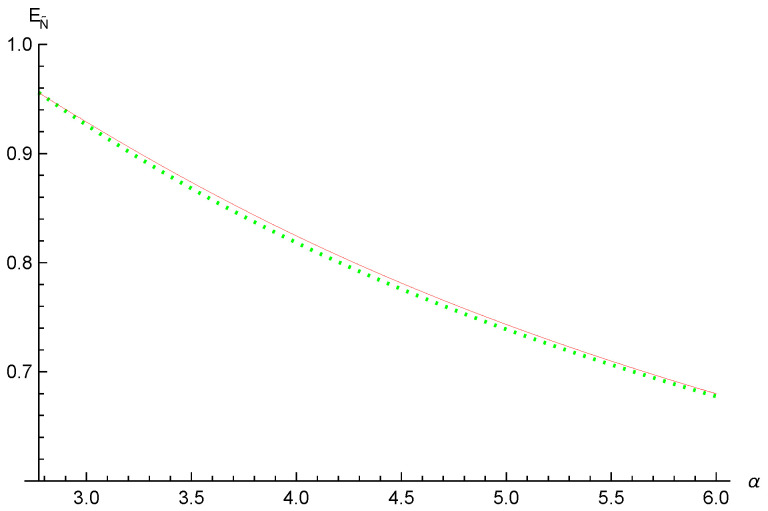
The red thin and green dotted lines depict the lower bounds from Equations (16) and (9), respectively.

*When $a=b=\frac{1}{\sqrt{2}}$, we have $\tilde{N}{(|\Phi \rangle }_{A|BCD})=1$, $\tilde{N}\left(\rho_{A|B}\right)=0.9107$, $\tilde{N}\left(\rho_{A|C}\right)=0.3333$ and $\tilde{N}\left(\rho_{A|D}\right)=0.244$. Using relation (2), we have $E_{\tilde{N}}\left(\rho_{A|BCD}\right)=1$, $E_{\tilde{N}}\left(\rho_{A|B}\right)=0.934101$, $E_{\tilde{N}}\left(\rho_{A|C}\right)=0.415001$ and $E_{\tilde{N}}\left(\rho_{A|D}\right)=0.314986$. Thus, ${\left[E_{\tilde{N}}\left(\rho_{A|BCD}\right)\right]}^{\alpha }\geq {\left(0.934101\right)}^{\alpha }+\frac{\alpha }{4\ln2}{\left(0.415001\right)}^{\alpha }+{\left(\frac{\alpha }{4\ln2}\right)}^{2}{\left(0.314986\right)}^{\alpha }$ from inequality (16), and ${\left[E_{\tilde{N}}\left(\rho_{A|BCD}\right)\right]}^{\alpha }\geq {\left(0.934101\right)}^{\alpha }+\frac{\alpha }{4\ln2}{\left(0.415001\right)}^{\alpha }+\frac{\alpha }{4\ln2}{\left(0.314986\right)}^{\alpha }$ from inequality (9). One can see that inequality (16) is better than inequality (9) for α≥4ln2, see Figure 2.*

We can also use the different examples to show that inequality (16) is better than inequality (9) for α≥4ln2.

**Example 3.**  *Let us consider the 4-qubit generalized W-class state,*

(19) $$\begin{array}{cc} {|W\rangle }_{ABCD} & =\lambda_{1}|1000\rangle +\lambda_{2}|0100\rangle \\ & \;\;\;\;+\lambda_{3}|0010\rangle +\lambda_{4}|0001\rangle , \end{array}$$

**Figure 3:**
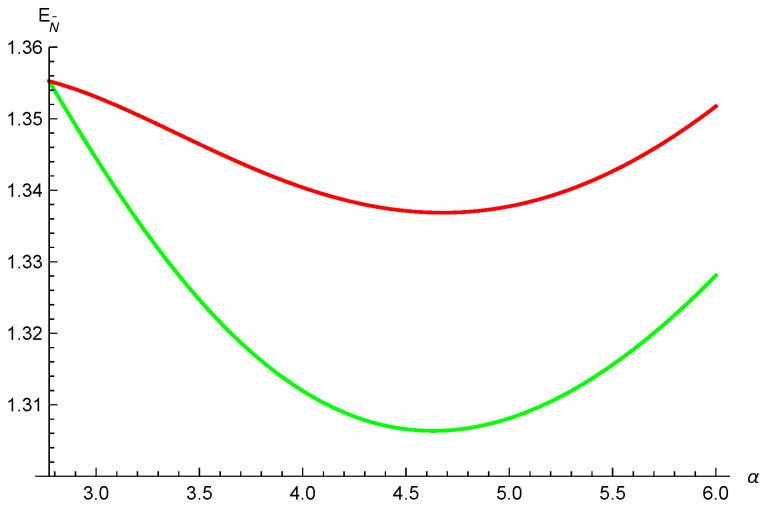
The red and green lines depict the lower bounds from Equations (16) and (9), respectively.

*where $\sum_{i}\lambda_{i}^{2}=1$. We have $\tilde{N}\left(\rho_{A|B}\right)=2\lambda_{1}\lambda_{2}$, $\tilde{N}\left(\rho_{A|C}\right)=2\lambda_{1}\lambda_{3}$ and $\tilde{N}\left(\rho_{A|D}\right)=2\lambda_{1}\lambda_{4}$. Taking $\lambda_{1}=\frac{3}{4}$, $\lambda_{2}=\frac{\sqrt{2}}{2}$, $\lambda_{3}=\frac{1}{4}$ and $\lambda_{4}=\frac{1}{4}$, we obtain $\tilde{N}\left(\rho_{A|B}\right)=\frac{3\sqrt{2}}{4}$, $\tilde{N}\left(\rho_{A|C}\right)=\frac{3}{8}$ and $\tilde{N}\left(\rho_{A|D}\right)=\frac{3}{8}$. Using relation (2), we have $E_{\tilde{N}}\left(\rho_{A|B}\right)=1.043$, $E_{\tilde{N}}\left(\rho_{A|C}\right)=0.459$ and $E_{\tilde{N}}\left(\rho_{A|D}\right)=0.459$. Thus, ${\left[E_{\tilde{N}}\left(\rho_{A|BCD}\right)\right]}^{\alpha }\geq {\left(1.043\right)}^{\alpha }+\frac{\alpha }{4\ln2}{\left(0.459\right)}^{\alpha }+{\left(\frac{\alpha }{4\ln2}\right)}^{2}{\left(0.459\right)}^{\alpha }$ from inequality (16), and ${\left[E_{\tilde{N}}\left(\rho_{A|BCD}\right)\right]}^{\alpha }\geq {\left(1.043\right)}^{\alpha }+\frac{\alpha }{4\ln2}{\left(0.459\right)}^{\alpha }+\frac{\alpha }{4\ln2}{\left(0.459\right)}^{\alpha }$ from inequality (9). One can see that inequality (16) is better than inequality (9) for α≥4ln2, see Figure 3.*

The conditions (17) are not universally fulfilled; we derive the subsequent monogamy inequality under alternate circumstances.

**Theorem 3.**  *For α≥4ln2, any (N+1)-qubit state $\rho_{AB_{0}\ldots B_{N-1}}$ satisfies*

(20) $$\begin{array}{ccc} {\left[E_{\tilde{N}}\left(\rho_{A|B_{0}\ldots B_{N-1}}\right)\right]}^{\alpha } & ⩾ & \sum_{j=0}^{t}{\left(\frac{\alpha }{4\ln2}\right)}^{j}{\left[E_{\tilde{N}}\left(\rho_{A|B_{j}}\right)\right]}^{\alpha }+{\left(\frac{\alpha }{4\ln2}\right)}^{t+2}\sum_{j=t+1}^{N-2}{\left[E_{\tilde{N}}\left(\rho_{A|B_{j}}\right)\right]}^{\alpha }\\ & & \;\;\;+{\left(\frac{\alpha }{4\ln2}\right)}^{t+1}{\left[E_{\tilde{N}}\left(\rho_{A|B_{N-1}}\right)\right]}^{\alpha }, \end{array}$$

*on the condition that $E_{\tilde{N}}^{4\ln2}\left(\rho_{A|B_{i}}\right)⩾E_{\tilde{N}}^{4\ln2}\left(\rho_{A|B_{i+1}\cdots B_{N-1}}\right)$ for i=0,1,⋯,t, and $E_{\tilde{N}}^{4\ln2}\left(\rho_{A|B_{j}}\right)⩽E_{\tilde{N}}^{4\ln2}\left(\rho_{A|B_{j+1}\cdots B_{N-1}}\right)$ for j=t+1,⋯,N−2, 0⩽t⩽N−3, N⩾3.*

**Proof.**  From Theorem 1 for the case N=2, we have

(21) $$\begin{array}{ccc} {\left[E_{\tilde{N}}\left(\rho_{A|B_{0}\ldots B_{N-1}}\right)\right]}^{\alpha } & ⩾ & {\left[E_{\tilde{N}}\left(\rho_{A|B_{0}}\right)\right]}^{\alpha }+\frac{\alpha }{4\ln2}{\left[E_{\tilde{N}}\left(\rho_{A|B_{1}\ldots B_{N-1}}\right)\right]}^{\alpha }\\ & ⩾ & \cdots \\ & ⩾ & \sum_{j=0}^{t}{\left(\frac{\alpha }{4\ln2}\right)}^{j}{\left[E_{\tilde{N}}\left(\rho_{A|B_{j}}\right)\right]}^{\alpha }+{\left(\frac{\alpha }{4\ln2}\right)}^{t+1}{\left[E_{\tilde{N}}\left(\rho_{A|B_{t}+1\ldots B_{N-1}}\right)\right]}^{\alpha }. \end{array}$$

Since $E_{\tilde{N}}^{4\ln2}\left(\rho_{A|B_{j}}\right)⩽E_{\tilde{N}}^{4\ln2}\left(\rho_{A|B_{j+1}\cdots B_{N-1}}\right)$ for j=t+1,⋯,N−2, using Theorem 1 again, we have

(22) $$\begin{array}{ccc} {\left[E_{\tilde{N}}\left(\rho_{A|B_{t}+1\ldots B_{N-1}}\right)\right]}^{\alpha } & ⩾ & \frac{\alpha }{4\ln2}{\left[E_{\tilde{N}}\left(\rho_{A|B_{t+1}}\right)\right]}^{\alpha }+{\left[E_{\tilde{N}}\left(\rho_{A|B_{t}+2\ldots B_{N-1}}\right)\right]}^{\alpha }\\ & ⩾ & \cdots \\ & ⩾ & \frac{\alpha }{4\ln2}\left(\sum_{j=t+1}^{N-2}{\left[E_{\tilde{N}}\left(\rho_{A|B_{j}}\right)\right]}^{\alpha }\right)+{\left[E_{\tilde{N}}\left(\rho_{A|B_{N-1}}\right)\right]}^{\alpha }. \end{array}$$

Combining (21) and (22), we obtain inequality (20). □

**Remark 3.**  *From Theorem 3, if $E_{\tilde{N}}^{4\ln2}\left(\rho_{A|B_{i}}\right)⩾E_{\tilde{N}}^{4\ln2}\left(\rho_{A|B_{i+1}\cdots B_{N-1}}\right)$ for all j=0,1,⋯,N−2, one has*

$$\begin{array}{c} {\left[E_{\tilde{N}}\left(\rho_{A|B_{0}B_{1}\ldots B_{N-1}}\right)\right]}^{\alpha }\geq \sum_{j=0}^{N-1}{\left(\frac{\alpha }{4\ln2}\right)}^{j}{\left[E_{\tilde{N}}\left(\rho_{A|B_{j}}\right)\right]}^{\alpha }. \end{array}$$

## 4. Tighter Polygamy Inequalities of Multi-Qubit LCRENoA

Here, we present refined polygamy inequalities by utilizing the αth-power of LCRENoA.

**Theorem 4.**  *For any (N+1)-qubit state $\rho_{AB_{0}\ldots B_{N-1}}$, we have*

(23) $${\left[E_{{\tilde{N}}_{a}}\left(\rho_{A|B_{0}B_{1}\ldots B_{N-1}}\right)\right]}^{\alpha }\leq \sum_{j=0}^{N-1}{\left(\frac{\alpha }{2}\right)}^{\omega_{H}\left(\vec{j}\right)}{\left[E_{{\tilde{N}}_{a}}\left(\rho_{A|B_{j}}\right)\right]}^{\alpha },$$

*where 0≤α≤2, $\vec{j}=\left(j_{0},\cdots ,j_{N-1}\right)$ is the vector from the binary representation of j, and $\omega_{H}\left(\vec{j}\right)$ is the Hamming weight of $\vec{j}$.*

**Proof.**  From inequality (5), one has $E_{{\tilde{N}}_{a}}^{2}\left(\rho_{A|B_{0}\cdots B_{N-1}}\right)\leq \sum_{i=0}^{N-1}E_{{\tilde{N}}_{a}}^{2}\left(\rho_{A|B_{i}}\right)$. Thus, it is sufficient to show that

(24) $${\left[\sum_{j=0}^{N-1}E_{{\tilde{N}}_{a}}^{2}\left(\rho_{A|B_{j}}\right)\right]}^{\frac{\alpha }{2}}\leq \sum_{j=0}^{N-1}{\left(\frac{\alpha }{2}\right)}^{\omega_{H}\left(\vec{j}\right)}{\left[E_{{\tilde{N}}_{a}}\left(\rho_{A|B_{j}}\right)\right]}^{\alpha }.$$

Assuming no loss of generality, we label the qubit subsystems $B_{0},\ldots ,B_{N-1}$ in a manner that preserves their intended order such that

(25) $$E_{{\tilde{N}}_{a}}^{2}\left(\rho_{A|B_{j}}\right)\geq E_{{\tilde{N}}_{a}}^{2}\left(\rho_{A|B_{j+1}}\right)\geq 0$$

for j=0,1,…,N−2.

Firstly, we demonstrate the validity of inequality (24) for the case of $N=2^{n}$. For n=1, we obtain

(26) $${\left[E_{{\tilde{N}}_{a}}^{2}\left(\rho_{A|B_{0}}\right)+E_{{\tilde{N}}_{a}}^{2}\left(\rho_{A|B_{1}}\right)\right]}^{\frac{\alpha }{2}}={\left[E_{{\tilde{N}}_{a}}\left(\rho_{A|B_{0}}\right)\right]}^{\alpha }{\left(1+\frac{E_{{\tilde{N}}_{a}}^{2}\left(\rho_{A|B_{1}}\right)}{E_{{\tilde{N}}_{a}}^{2}\left(\rho_{A|B_{0}}\right)}\right)}^{\frac{\alpha }{2}}.$$

Combining (8) and (25), we have

(27) $${\left(1+\frac{E_{{\tilde{N}}_{a}}^{2}\left(\rho_{A|B_{1}}\right)}{E_{{\tilde{N}}_{a}}^{2}\left(\rho_{A|B_{0}}\right)}\right)}^{\frac{\alpha }{2}}\leq 1+\frac{\alpha }{2}{\left(\frac{E_{{\tilde{N}}_{a}}\left(\rho_{A|B_{1}}\right)}{E_{{\tilde{N}}_{a}}\left(\rho_{A|B_{0}}\right)}\right)}^{\alpha }.$$

From (26) and (27), we obtain

$${\left[E_{{\tilde{N}}_{a}}^{2}\left(\rho_{A|B_{0}}\right)+E_{{\tilde{N}}_{a}}^{2}\left(\rho_{A|B_{1}}\right)\right]}^{\frac{\alpha }{2}}\leq {\left[E_{{\tilde{N}}_{a}}\left(\rho_{A|B_{0}}\right)\right]}^{\alpha }+\frac{\alpha }{2}{\left[E_{{\tilde{N}}_{a}}\left(\rho_{A|B_{1}}\right)\right]}^{\alpha }.$$

Therefore, inequality (24) holds for n=1.

Taking into consideration that inequality (24) has already been established for the case where $N=2^{n-1}$, with n≥2, we now proceed to demonstrate its validity for the case of $N=2^{n}$, and we have

(28) $$\begin{array}{c} {\left(\sum_{j=0}^{N-1}E_{{\tilde{N}}_{a}}^{2}\left(\rho_{A|B_{j}}\right)\right)}^{\frac{\alpha }{2}}={\left(\sum_{j=0}^{2^{n-1}-1}E_{{\tilde{N}}_{a}}^{2}\left(\rho_{A|B_{j}}\right)\right)}^{\frac{\alpha }{2}}{\left(1+\frac{\sum_{j=2^{n-1}}^{2^{n}-1}E_{{\tilde{N}}_{a}}^{2}\left(\rho_{A|B_{j}}\right)}{\sum_{j=0}^{2^{n-1}-1}E_{{\tilde{N}}_{a}}^{2}\left(\rho_{A|B_{j}}\right)}\right)}^{\frac{\alpha }{2}}. \end{array}$$

Because of the ordering of subsystems, inequality (25) implies

$$0\leq \frac{\sum_{j=2^{n-1}}^{2^{n}-1}E_{{\tilde{N}}_{a}}^{2}\left(\rho_{A|B_{j}}\right)}{\sum_{j=0}^{2^{n-1}-1}E_{{\tilde{N}}_{a}}^{2}\left(\rho_{A|B_{j}}\right)}\leq 1.$$

Thus, Equation (28) and inequality (8) lead to

$${\left(\sum_{j=0}^{N-1}E_{{\tilde{N}}_{a}}^{2}\left(\rho_{A|B_{j}}\right)\right)}^{\frac{\alpha }{2}}\leq {\left(\sum_{j=0}^{2^{n-1}-1}E_{{\tilde{N}}_{a}}^{2}\left(\rho_{A|B_{j}}\right)\right)}^{\frac{\alpha }{2}}+\frac{\alpha }{2}{\left(\sum_{j=2^{n-1}}^{2^{n}-1}E_{{\tilde{N}}_{a}}^{2}\left(\rho_{A|B_{j}}\right)\right)}^{\frac{\alpha }{2}}.$$

By the induction hypothesis, we obtain

$${\left(\sum_{j=0}^{2^{n-1}-1}E_{{\tilde{N}}_{a}}^{2}\left(\rho_{A|B_{j}}\right)\right)}^{\frac{\alpha }{2}}\leq \sum_{j=0}^{2^{n-1}-1}{\left(\frac{\alpha }{2}\right)}^{\omega_{H}\left(\vec{j}\right)}{\left[E_{{\tilde{N}}_{a}}\left(\rho_{A|B_{j}}\right)\right]}^{\alpha }.$$

Through the process of reassigning labels to the subsystems, the induction hypothesis offers the following outcome,

$${\left(\sum_{j=2^{n-1}}^{2^{n}-1}E_{{\tilde{N}}_{a}}^{2}\left(\rho_{A|B_{j}}\right)\right)}^{\frac{\alpha }{2}}\leq \sum_{j=2^{n-1}}^{2^{n}-1}{\left(\frac{\alpha }{2}\right)}^{\omega_{H}\left(\vec{j}\right)-1}{\left[E_{{\tilde{N}}_{a}}\left(\rho_{A|B_{j}}\right)\right]}^{\alpha }.$$

Thus, we have

$${\left(\sum_{j=0}^{2^{n}-1}E_{{\tilde{N}}_{a}}^{2}\left(\rho_{A|B_{j}}\right)\right)}^{\frac{\alpha }{2}}\leq \sum_{j=0}^{2^{n}-1}{\left(\frac{\alpha }{2}\right)}^{\omega_{H}\left(\vec{j}\right)}{\left[E_{{\tilde{N}}_{a}}\left(\rho_{A|B_{j}}\right)\right]}^{\alpha }.$$

Now, consider a $\left(2^{n}+1\right)$-qubit state $\Gamma_{AB_{0}B_{1}\ldots B_{2^{n}-1}}=\rho_{AB_{0}B_{1}\ldots B_{N-1}}⊗\sigma_{B_{N}\ldots B_{2^{n}-1}}$. We have

$${\left[E_{{\tilde{N}}_{a}}^{2}\left(\Gamma_{A|B_{0}B_{1}\ldots B_{2^{n}-1}}\right)\right]}^{\frac{\alpha }{2}}\leq \sum_{j=0}^{2^{n}-1}{\left(\frac{\alpha }{2}\right)}^{\omega_{H}\left(\vec{j}\right)}{\left[E_{{\tilde{N}}_{a}}\left(\Gamma_{A|B_{j}}\right)\right]}^{\alpha },$$

Therefore,

$$\begin{array}{cc} {\left[E_{{\tilde{N}}_{a}}^{2}\left(\rho_{A|B_{0}B_{1}\ldots B_{N-1}}\right)\right]}^{\frac{\alpha }{2}}= & {\left[E_{{\tilde{N}}_{a}}^{2}\left(\Gamma_{A|B_{0}B_{1}\ldots B_{2^{n}-1}}\right)\right]}^{\frac{\alpha }{2}}\\ \leq & \sum_{j=0}^{2^{n}-1}{\left(\frac{\alpha }{2}\right)}^{\omega_{H}\left(\vec{j}\right)}{\left[E_{{\tilde{N}}_{a}}\left(\Gamma_{A|B_{j}}\right)\right]}^{\alpha }\\ = & \sum_{j=0}^{N-1}{\left(\frac{\alpha }{2}\right)}^{\omega_{H}\left(\vec{j}\right)}{\left[E_{{\tilde{N}}_{a}}\left(\rho_{A|B_{j}}\right)\right]}^{\alpha }, \end{array}$$

where $E_{{\tilde{N}}_{a}}\left(\Gamma_{A|B_{0}B_{1}\cdots B_{2^{n}-1}}\right)=E_{{\tilde{N}}_{a}}\left(\rho_{A|B_{0}B_{1}\cdots B_{N-1}}\right)$, $E_{{\tilde{N}}_{a}}\left(\Gamma_{A|B_{j}}\right)=0$ for $j=N,\cdots ,2^{n}-1$, and $\Gamma_{A|B_{j}}=\rho_{A|B_{j}}$ for each j=0,⋯,N−1, which completes the proof. □

**Remark 4.**  *Since ${\left(\frac{\alpha }{2}\right)}^{\omega_{H}\left(\vec{j}\right)}\leq 1$ for any 0≤α≤2, for any (N+1)-qubit state $\rho_{AB_{0}B_{1}\cdots B_{N-1}}$, we have the following relation*

$${\left[E_{{\tilde{N}}_{a}}\left(\rho_{A|B_{0}B_{1}\ldots B_{N-1}}\right)\right]}^{\alpha }\leq \sum_{j=0}^{N-1}{\left(\frac{\alpha }{2}\right)}^{\omega_{H}\left(\vec{j}\right)}{\left[E_{{\tilde{N}}_{a}}\left(\rho_{A|B_{j}}\right)\right]}^{\alpha }\leq \sum_{j=0}^{N-1}{\left[E_{{\tilde{N}}_{a}}\left(\rho_{A|B_{j}}\right)\right]}^{\alpha }.$$

*Therefore, our inequality (23) in Theorem 4 is always tighter than inequality (5) in Ref. [36].*

**Example 4.**  *Let us consider the 3-qubit generalized W state,*

$${|W\rangle }_{ABC}=\frac{1}{\sqrt{3}}(|100\rangle +|010\rangle +|001\rangle ).$$

**Figure 4:**
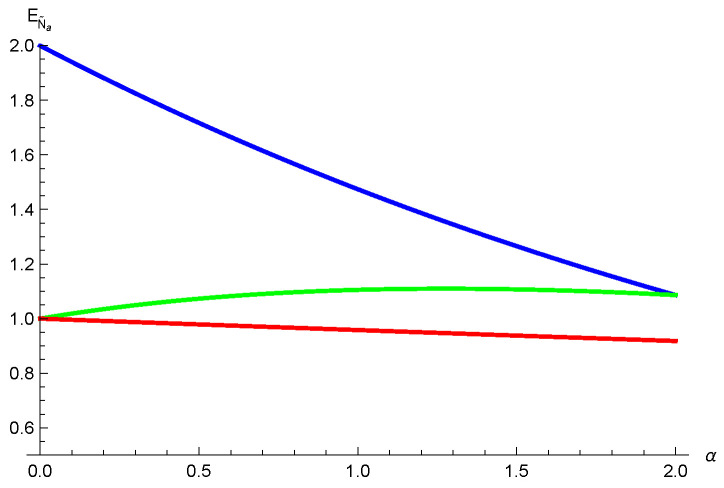
The red line is the exact value of $E_{{\tilde{N}}_{a}}\left(\rho_{A|BC}\right)$. The green line represents the upper bound from our result (23). The blue line represents the upper bound from the result (5) in [36].

*We have ${\tilde{N}}_{a}\left(\rho_{A|BC}\right)=2\sqrt{2}/3$, ${\tilde{N}}_{a}\left(\rho_{A|B}\right)=2/3$ and ${\tilde{N}}_{a}\left(\rho_{A|C}\right)=2/3$. Using relation (4) we have $E_{{\tilde{N}}_{a}}\left(\rho_{A|BC}\right)=\log_{2}\left(2\sqrt{2}/3+1\right)$, $E_{{\tilde{N}}_{a}}\left(\rho_{A|B}\right)=\log_{2}\frac{5}{3}$ and $E_{{\tilde{N}}_{a}}\left(\rho_{A|C}\right)=\log_{2}\frac{5}{3}$. Thus, ${\left[E_{{\tilde{N}}_{a}}\left(\rho_{A|BC}\right)\right]}^{\alpha }\leq {\left(\log_{2}\frac{5}{3}\right)}^{\alpha }+\frac{\alpha }{2}{\left(\log_{2}\frac{5}{3}\right)}^{\alpha }$ from our result (23), and ${\left[E_{{\tilde{N}}_{a}}\left(\rho_{A|BC}\right)\right]}^{\alpha }\leq (\log_{2}{\left(\log_{2}\frac{5}{3}\right)}^{\alpha }+{\left(\log_{2}\frac{5}{3}\right)}^{\alpha }$ from the result (5) given in Ref. [36]. One can see that our result (23) is better than the result (5) in Ref. [36] for 0≤α≤2, see Figure 4.*

Just as the transition from inequality (9) to inequality (16), we can likewise enhance the polygamy inequality in Theorem 4. The proof is similar to Theorem 2.

**Theorem 5.**  *For 0≤α≤2, any (N+1)-qubit state $\rho_{AB_{0}\ldots B_{N-1}}$ satisfies*

(29) $${\left[E_{{\tilde{N}}_{a}}\left(\rho_{A|B_{0}B_{1}\ldots B_{N-1}}\right)\right]}^{\alpha }\leq \sum_{j=0}^{N-1}{\left(\frac{\alpha }{2}\right)}^{j}{\left[E_{{\tilde{N}}_{a}}\left(\rho_{A|B_{j}}\right)\right]}^{\alpha },$$

*if*

$$E_{{\tilde{N}}_{a}}^{2}\left(\rho_{A|B_{i}}\right)\geq \sum_{j=i+1}^{N-1}E_{{\tilde{N}}_{a}}^{2}\left(\rho_{A|B_{j}}\right)$$

*for i=0,1,…,N−2.*

**Remark 5.**  *In fact, according to relation (6), for any 0≤α≤2, one has*

$$\begin{array}{cc} {\left[E_{{\tilde{N}}_{a}}\left(\rho_{A|B_{0}\ldots B_{N-1}}\right)\right]}^{\alpha }\leq & \sum_{j=0}^{N-1}{\left(\frac{\alpha }{2}\right)}^{j}{\left(E_{{\tilde{N}}_{a}}\left(\rho_{A|B_{j}}\right)\right)}^{\alpha }\\ \leq & \sum_{j=0}^{N-1}{\left(\frac{\alpha }{2}\right)}^{\omega_{H}\left(\vec{j}\right)}{\left[E_{{\tilde{N}}_{a}}\left(\rho_{A|B_{j}}\right)\right]}^{\alpha }. \end{array}$$

*Therefore, inequality (29) in Theorem 6 is tighter than inequality (23) of Theorem 5 under certain conditions.*

**Figure 5:**
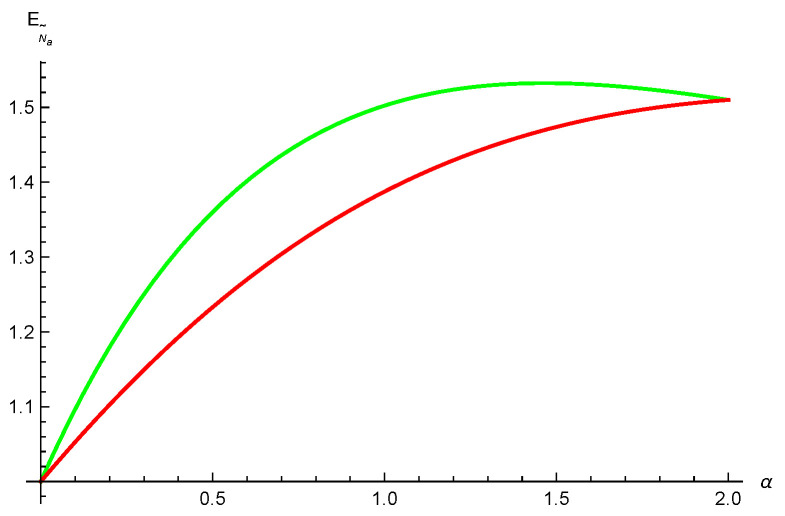
The red and green lines depict the lower bounds from Equations (29) and (23), respectively.

**Example 5.**  *Let us consider the 4-qubit generalized W-class state again in Example 3. We have ${\tilde{N}}_{a}\left(\rho_{A|B}\right)=2\lambda_{1}\lambda_{2}$, ${\tilde{N}}_{a}\left(\rho_{A|C}\right)=2\lambda_{1}\lambda_{3}$ and ${\tilde{N}}_{a}\left(\rho_{A|D}\right)=2\lambda_{1}\lambda_{4}$. Taking $\lambda_{1}=\frac{3}{4},\;\lambda_{2}=\frac{\sqrt{2}}{2},\;\lambda_{3}=\frac{1}{4}$ and $\lambda_{4}=\frac{1}{4}$, we obtain ${\tilde{N}}_{a}\left(\rho_{A|B}\right)=\frac{3\sqrt{2}}{4}$, ${\tilde{N}}_{a}\left(\rho_{A|C}\right)=\frac{3}{8}$ and ${\tilde{N}}_{a}\left(\rho_{A|D}\right)=\frac{3}{8}$. Using relation (4), we have $E_{{\tilde{N}}_{a}}\left(\rho_{A|B}\right)=1.043$, $E_{{\tilde{N}}_{a}}\left(\rho_{A|C}\right)=0.459$ and $E_{{\tilde{N}}_{a}}\left(\rho_{A|D}\right)=0.459$. Thus, ${\left[E_{{\tilde{N}}_{a}}\left(\rho_{A|BCD}\right)\right]}^{\alpha }\leq {\left(1.043\right)}^{\alpha }+\frac{\alpha }{2}{\left(0.459\right)}^{\alpha }+{\left(\frac{\alpha }{2}\right)}^{2}{\left(0.459\right)}^{\alpha }$ from inequality (29), and ${\left[E_{{\tilde{N}}_{a}}\left(\rho_{A|BCD}\right)\right]}^{\alpha }\leq {\left(1.043\right)}^{\alpha }+\frac{\alpha }{2}{\left(0.459\right)}^{\alpha }+\frac{\alpha }{2}{\left(0.459\right)}^{\alpha }$ from inequality (23). One can see that inequality (29) is better than inequality (23) for 0≤α≤2, see Figure 5.*

By modifying the conditions stated in Theorem 5, we are able to present more comprehensive results.

**Theorem 6.**  *For 0≤α≤2, any (N+1)-qubit state $\rho_{AB_{0}\ldots B_{N-1}}$ satisfies*

$$\begin{array}{ccc} {\left[E_{{\tilde{N}}_{a}}\left(\rho_{A|B_{0}\ldots B_{N-1}}\right)\right]}^{\alpha } & \leq & \sum_{j=0}^{t}{\left(\frac{\alpha }{2}\right)}^{j}{\left[E_{{\tilde{N}}_{a}}\left(\rho_{A|B_{j}}\right)\right]}^{\alpha }+{\left(\frac{\alpha }{2}\right)}^{t+2}\sum_{j=t+1}^{N-2}{\left[E_{{\tilde{N}}_{a}}\left(\rho_{A|B_{j}}\right)\right]}^{\alpha }\\ & & \;\;\;+{\left(\frac{\alpha }{2}\right)}^{t+1}{\left[E_{{\tilde{N}}_{a}}\left(\rho_{A|B_{N-1}}\right)\right]}^{\alpha }, \end{array}$$

*on the condition that $E_{{\tilde{N}}_{a}}^{2}\left(\rho_{A|B_{i}}\right)⩾E_{{\tilde{N}}_{a}}^{2}\left(\rho_{A|B_{i+1}\cdots B_{N-1}}\right)$ for i=0,1,⋯,t and $E_{{\tilde{N}}_{a}}^{2}\left(\rho_{A|B_{j}}\right)⩽E_{{\tilde{N}}_{a}}^{2}\left(\rho_{A|B_{j+1}\cdots B_{N-1}}\right)$ for j=t+1,⋯,N−2, 0⩽t⩽N−3, N⩾3.*

**Remark 6.**  *From Theorem 6, if $E_{{\tilde{N}}_{a}}^{2}\left(\rho_{A|B_{i}}\right)⩾E_{{\tilde{N}}_{a}}^{2}\left(\rho_{A|B_{i+1}\cdots B_{N-1}}\right)$ for all j=0,1,⋯,N−2, we have*

$${\left[E_{{\tilde{N}}_{a}}\left(\rho_{A|B_{0}B_{1}\ldots B_{N-1}}\right)\right]}^{\alpha }\leq \sum_{j=0}^{N-1}{\left(\frac{\alpha }{2}\right)}^{j}{\left[E_{{\tilde{N}}_{a}}\left(\rho_{A|B_{j}}\right)\right]}^{\alpha }.$$

## 5. Conclusions and Discussions

Monogamy and polygamy relations exemplify the fundamental properties displayed in multi-qubit entanglement, exhibiting the intricate nature of quantum entanglement. We elucidated the manifestations of multi-qubit monogamy and polygamy constraints by utilizing the nonconvex entanglement measures LCREN and LCRENoA. We integrated the Hamming weight and the LCREN (LCRENoA) for the first time, and presented new classes of monogamy and polygamy relations. We also demonstrated that these new inequalities impose finer constraints than the previous ones. Our approaches may be used in future studies aimed at comprehending the entanglement distribution in multi-qubit systems.

We focused on multi-qubit systems. It is noteworthy that our tight monogamy inequality (9) remains applicable not only to such systems but also to certain higher-dimensional quantum systems for which the CKW monogamy inequality (1) is violated. First, let us recall the definition of tangle. The tangle of a bipartite pure state ${|\psi \rangle }_{AB}$ is defined as $\tau {(|\psi \rangle }_{A|B})=2\left(1-\mathrm{tr}\rho_{A}^{2}\right)$, where $\rho_{A}={\mathrm{tr}}_{B}{|\psi \rangle }_{AB}\langle \psi |$ [12]. The tangle of a bipartite mixed state $\rho_{AB}$ is defined by [12]

$$\tau \left(\rho_{A|B}\right)={\left[\min_{\left\{p_{k},|\psi_{k}\rangle \right\}}\sum_{k}p_{k}\sqrt{\tau {(|\psi_{k}\rangle }_{A|B})}\right]}^{2},$$

where the minimization is taken over all possible pure-state decompositions of $\rho_{AB}=\sum_{k}p_{k}|\psi_{k}\rangle_{AB}\langle \psi_{k}|$ with $p_{k}\geq 0$, and $\sum_{k}p_{k}=1$. For multi-qubit states, the tangle satisfies the following monogamy inequality,

(30) $$\tau \left(\rho_{A|B_{0}B_{1}\cdots A_{N}-1}\right)\geq \sum_{j=0}^{N-1}\tau \left(\rho_{A|B_{j}}\right).$$

Nevertheless, the monogamy inequality (30) based on the tangle does not generally hold for systems with higher dimensions [14,42,43]. Specifically, one can readily confirm that the following 3⊗3⊗3 three-qutrit state violates the inequality (30),

(31) $$\begin{array}{ccc} {|\Psi \rangle }_{A|BC} & = & \frac{1}{\sqrt{6}}(|012\rangle -|021\rangle +|120\rangle -|102\rangle +|201\rangle -|210\rangle ). \end{array}$$

In addition, the following 3⊗2⊗2 state also violates the inequality (30),

(32) $$\begin{array}{ccc} {|\Psi \rangle }_{ABC} & = & \frac{1}{\sqrt{6}}(\sqrt{2}|010\rangle +\sqrt{2}|101\rangle +|200\rangle +|211\rangle ). \end{array}$$

**Figure 6:**
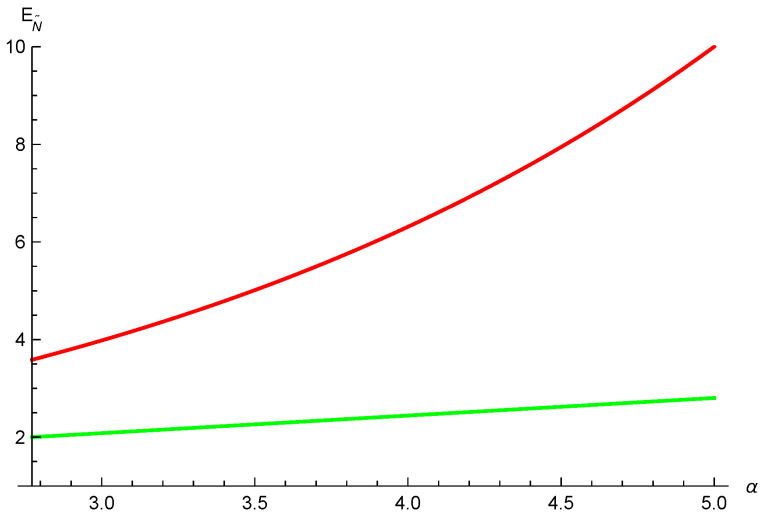
The red line is the exact value of $E_{\tilde{N}}{(|\Psi \rangle }_{A|BC})$. The green line represents the lower bound from our results (9).

Concerning our LCREN-based monogamy inequality (9) for the quantum state (31), we have $\tilde{N}{(|\Psi \rangle }_{A|BC})=2$ and $\tilde{N}\left(\rho_{A|B}\right)=\tilde{N}\left(\rho_{A|C}\right)=1$. Using relation (2), we have $E_{\tilde{N}}{(|\Psi \rangle }_{A|BC})=\log_{2}3$ and $E_{\tilde{N}}\left(\rho_{A|B}\right)=E_{\tilde{N}}\left(\rho_{A|C}\right)=1$. Thus, we have

$$E_{\tilde{N}}^{\alpha }{(|\Psi \rangle }_{A|BC})={\left(\log_{2}3\right)}^{\alpha }\geq 1+\frac{\alpha }{4\ln2}=E_{\tilde{N}}^{\alpha }\left(\rho_{A|B}\right)+\frac{\alpha }{4\ln2}E_{\tilde{N}}^{\alpha }\left(\rho_{A|C}\right)$$

for α≥4ln2. Namely, our monogamy inequality (9) still holds for state (31), see Figure 6.

Likewise, considering the quantum state (32), we have $\tilde{N}{(|\Psi \rangle }_{A|BC})=2$ and $\tilde{N}\left(\rho_{A|B}\right)=\tilde{N}\left(\rho_{A|C}\right)=\frac{2\sqrt{2}}{3}$. Using relation (2), we have $E_{\tilde{N}}{(|\Psi \rangle }_{A|BC})=\log_{2}3$ and $E_{\tilde{N}}\left(\rho_{A|B}\right)=E_{\tilde{N}}\left(\rho_{A|C}\right)=\log_{2}\left(\frac{2\sqrt{2}}{3}+1\right)$. Thus, we have

$$E_{\tilde{N}}^{\alpha }{(|\Psi \rangle }_{A|BC})=\left(\log_{2}3^{\alpha }\right)\geq \left(1+\frac{\alpha }{4\ln2}\right){\left(\log_{2}\left(\frac{2\sqrt{2}}{3}+1\right)\right)}^{\alpha }=E_{\tilde{N}}^{\alpha }\left(\rho_{A|B}\right)+\frac{\alpha }{4\ln2}E_{\tilde{N}}^{\alpha }\left(\rho_{A|C}\right)$$

for α≥4ln2. In other words, The LCREN-based monogamy inequality (9) remains applicable to high-dimensional states that violate the CKW monogamy inequality (1). Our discoveries may shed light on further investigations on entanglement distribution in high-dimensional systems.

## Data Availability

All data generated or analyzed during this study are included and cited in this article.
